# [Retracted] MicroRNA‑154 inhibits the growth and metastasis of gastric cancer cells by directly targeting MTDH

**DOI:** 10.3892/ol.2023.13673

**Published:** 2023-01-17

**Authors:** Wenhui Qiao, Nong Cao, Lei Yang

Oncol Lett 14: 3268–3274, 2017; DOI: 10.3892/ol.2017.6558

Following the publication of this paper, it was drawn to the Editor's attention by a concerned reader that certain of the cell invasion and migration assay data shown in Figs. 2C and 4C, and western blotting data shown in Fig. 3D were strikingly similar to data appearing in different form in several other articles by different authors at different research institutes, some of which have been retracted. Owing to the fact that the contentious data in the above article had already been published, or were already under consideration for publication, elsewhere prior to its submission to *Oncology Letters*, the Editor has decided that this paper should be retracted from the Journal. The authors were asked for an explanation to account for these concerns, but the Editorial Office did not receive a reply. The Editor apologizes to the readership for any inconvenience caused.

